# Subtypes of Depression: Latent Class Analysis in Spanish Old People with Depressive Symptoms

**DOI:** 10.3390/life10050070

**Published:** 2020-05-18

**Authors:** Sergio Pérez-Belmonte, Laura Galiana, Patricia Sancho, Amparo Oliver, José M. Tomás

**Affiliations:** 1 Department of Methodology for the Behavioral Sciences, University of Valencia, 46010 Valencia, Spain; Sergioperezbel@gmail.com (S.P.-B.); oliver@uv.es (A.O.); tomasjm@uv.es (J.M.T.); 2 Department of Educational and Developmental Psychology, University of Valencia, 46010 Valencia, Spain; Patricia.Sancho@uv.es

**Keywords:** major depressive disorder, elderly, quality of life

## Abstract

Major depressive disorder (MDD) is one of the most disabling disorders and the one that most contributes to disability. When it occurs in older people, it is an additional burden to their potential physical and cognitive deficiencies, making MDD an important public health problem that supposes a large investment in health. There is a clear lack of consistency between the subtypes of depression found in the literature, ranging from two to seven classes, with three being the most commonly found non-melancholic, melancholic and psychotic, or putative psychotics. The aim of this research is to add knowledge to the profiles of depressive symptoms in a representative sample of older Spanish people, and to study the possible relationship of these symptom profiles with variables that have traditionally been related to depression. Spanish data from the sixth wave of SHARE were used, with 612 Spanish older adults living in Spain. A routine of several LCAs with a different number of classes was performed to answer this first aim to classify Spanish adults with depression symptoms. The results pointed out the presence of three different classes among the participants in the study: psychosomatic (11.12%), melancholic (14.21%), and anhedonic (74.67%). This work represents a step forward to understand the heterogeneity of major depressive disorder, facilitating the diagnosis, and subsequent treatment of older adults.

## 1. Introduction

Major depressive disorder (MDD) is one of the most disabling disorders worldwide [1], being the one that most contributes to disability, with a 7.5% global average (WHO, 2017) representing 4.3% of the global disease burden [2] MDD has a prevalence of 14% among people 65 years or older [3,4]. Older adults generally do not seek treatment [5] which, added to their potential physical and cognitive deficiencies, makes MDD an important public health problem that supposes a large investment in health. Specifically in Spain, this investment is around 1% of the GDP or 10,763 millions of euros [6].

A diagnostic of MDD in this population may be complex, given the characteristics such as increased somatization, masking symptoms, confusion with frequent life situations (bereavement, changes of address, loss of physical and mental abilities), or difficulty in making a differential diagnosis with dementia [7]. For a deeper understanding of the disorder and its heterogeneity in old age, recent research has been collecting evidence of clinically relevant depression subtypes [8]. For example, Sneed, J.R., et al found two types of depression, vascular and non-vascular depression [9], in two different samples of community-dwelling old people with a diagnosed previous major depressive episode. Hybels, C.F., et al found three latent classes for depressive symptoms in a longitudinal study of 4000 older adults from the general population: low presence of symptoms; negative affect and somatic symptoms; and high presence of all symptoms [5]. The research by [10] with 400 people 65 years or older without symptoms of dementia and one or more depressive symptoms also found three classes: minor depression, major depression, and major or minor depression with psychomotor symptoms.

Given that studies in old people are scarce, it is worth noting some research outcomes found in the adult population. [11] distinguished two classes or subtypes of depression: nuclear and non-nuclear. The authors in [12,13] found different clusters: non-melancholic and melancholic depression [12]; melancholic non-psychotic depression and melancholic psychotic depression [14]; and non-melancholic and melancholic depressed people [13]. These classes were summarized into three in subsequent works, in which they distinguished between: non-melancholic, melancholic and psychotic [15], or putative psychotics [16]. Along this line, other studies have also found three classes or clusters, as the work by [17] (moderate depression, cognitive-affective affliction, and severe depression); [18] (severe melancholic, severe atypical, and moderate severity); [19] (slow response, immediate response, and rapid response); or [20] (typical severe depression, severe atypical, and moderate depression).

Other studies have found four clusters of symptoms. For example, [21], in a sample of 12,180 people from the National Epidemiologic Survey on Alcohol and Related Conditions (NESARC) study wave 1 (2001–2002) found evidence of no depression, psychosomatic depression, cognitive-emotional symptoms, and severe depression.. [22], in a sample of 13,424 people from the USA who met MDD criteria A of the DSM-IV [23] selected from the NESARC wave 1 (2001–2002), found a four-class model: mild, cognitive, psychosomatic, and severe. Likewise, [24] found in a sample of 2772 people with moderate or severe depression and without psychotic symptoms, evidence of mild depression, moderate, severe with increased appetite, and severe with insomnia. [25], meanwhile, distinguished between mild depression, moderate depression without anxiety, moderate depression with anxiety, and severe depression with anxiety.

There are studies that increase the number of classes to six or seven. [26], for example, found a six-class model: less typical, typical mild, mild atypical, intermediate, severe typical, and severe atypical. [27], meanwhile, found a seven-class model: less typical, typical, atypical, non-appetitive, mood only, binge eating, and agitated. Therefore, the number of subtypes of depression found varies from two to seven, with three being the most commonly found. 

Among the subtypes of depression detected, three have been the most commonly found: severe, melancholic, and moderate depression. With regard to severe depression, this category has received different names. For example, [5] pointed out the existence of a class with the presence of all the symptoms. [17,21], and [22] specifically referred to the severe depression type. Other authors such as [20,26,28] clustered similar symptomatology under the class severe-typical depression; and in other studies, the severe depression subtype was characterized with specific symptoms such as anxiety [25] or insomnia [24]. The melancholic subtype of depression was found in [11] (although named after nuclear depression), [12,13,14,15,16,18]. Finally, a class of depression characterized by a moderate symptomatology was found in [17,18,20,24,25,26,28].

As [8] highlighted, there is a clear lack of consistency between the subtypes of depression found in the literature. This is largely due to the different criteria used to distinguish the classes, which include the severity of depression, individual symptoms that show the likelihood of response to exceptionally high items, the degree of agreement with the characteristics of the DSM-5 of atypical and melancholic depression [8], or the absence of biological measures to derive the stratifications [29].

Some of the studies that have been mentioned relate these subtypes with sociodemographic variables with diverse results. For example, [5] found, on one hand, that individuals in the class with a low presence of symptoms were characterized by being younger, male, married, presenting less cognitive impairment and functional limitations as well as a lower probability of having had negative life events in the previous year, and better physical health compared to those in the class of negative affect and somatic symptoms and the class with a high presence of all symptoms. On the other hand, they observed that the class with a high presence of all symptoms were those of older age, female, had fewer years of education, were not married, had worse health and more chronic diseases, more functional limitations, less perceived social support, and more stressful experiences. [30], on the other hand, observed that women were at greater risk of being in the severe atypical class and that they exhibited more transitions between classes, particularly with respect to the severe subtype, than men. Along these same lines, [18] also found that there were more women in the atypical type. However, the results of [10] found no predictive ability of age, sex, education, marital status, early or late onset, number of episodes, duration of the episode, and functional status for any of the three classes.

Other variables that have traditionally been related to MDD are being female [31,32]; being older [33]; marital status, with greater vulnerability in widowed older adults [34]; having lower levels of physical health [31] or greater number of chronic diseases [35]; having mobility problems [34]; performing little or no physical exercise [36]; and having a smaller social network [34]. In addition, there is a significant association between the severity of depression and poorer quality of life in old age, according to the review by [37].

In conclusion, and taking into account the heterogeneity of studies on depression as well as the importance of a successful diagnosis and treatment given its morbidity, mortality, and high prevalence, the objective of this research was to learn the profiles of depressive symptoms in a representative sample of older Spanish people, and study the possible relationship of these symptom profiles with variables that have traditionally been related to depression.

## 2. Materials and Methods

### 2.1. Design, Procedure, and Participants

The study relies on data from SHARE Wave 6 [38,39]. That is, we used the sixth measurement (wave) made since the beginning of the survey. The SHARE project is a longitudinal study aimed at assessing the population aged 50 and older and their spouses/partners across countries by using probability-based sampling [40]. The sampling protocol consists of a four-stage process: (1) drawing of a baseline or refreshment sample from each country, which has to provide a Sample Design Form regarding the sampling frame and associated sampling design; (2) evaluation and approval of the sampling proposal by the SHARE Central coordination; (3) sample drawing according to the approved sample design process; and (4) a Gross Sample File is provided by each country. Therefore, SHARE offers representative national (and European) data for all countries involved.

This study was a secondary data analysis; therefore, no research ethics approval was needed. However, we strictly followed the RESPECT Code of Practice for Socio-Economic Research [41], together with the WMA Declaration of Helsinki – Ethical Principles for Medical Research Involving Human Subjects [42].

For our purposes, only Spanish data from the sixth wave of SHARE were used. The inclusion criteria were: (a) being 60 years old or older; and (b) the presence of symptoms compatible with clinical depression. In order to diagnose the presence of symptoms compatible with clinical depression, a cut-off point of six (presence of at least six symptoms) was used in the European Union initiative to compare the symptoms of depression (EURO-D). The resulting sample for this research consisted of 612 Spanish older adults residing in Spain. Of these, 72.2% (n = 442) were women and 27.8% (n = 170) were men. The average age was 76.13 years (SD = 8.95).

### 2.2. Measurement Instruments

For the analyses of latent classes in depression, the EURO-D scale was used [43]. The scale consists of 12 binary items that indicate the presence or absence during the last month of: depressed mood, pessimism/hopelessness, suicidal thoughts, feelings of guilt, irritability, tears, fatigue, sleep disorders, loss of interest, changes in appetite, concentration problems, and lack of pleasure. The total score varies from 0 to 12, with a direct relationship between score and number of symptoms, with the cut-off point for depression equal to or greater than four (in this work, a cut-off point of six or more was chosen to ensure that the individuals had major depressive disorder, as previously mentioned). The EURO-D is the instrument included in SHARE to assess depression. It was originally developed to harmonize data on late-life depression from population studies in 11 European countries [43].

Regarding the sociodemographic variables measured in SHARE, we included age, gender, marital status (widowers vs. non-widowers), and coexistence (with vs. without a partner). In addition, indicators of perceived health, physical activity, social support, and quality of life were also used. Health was measured with the item of the Short Form-36 Health Survey (SF-36) [44] regarding general health, namely “Would you say your health is…?”. The indicator scores on a 5-point Likert scale ranged from 1 (poor) to 5 (excellent). An indicator of the number of chronic illness was also measured, with values ranging from 0 to 8. Mobility problems were assessed with one indicator from 0 “no mobility problems” to 4 “many mobility problems”. With regard to social support, it was measured with the frequency that the person received help from others (outside home) with a yes/no answer. Physical activity was assessed by asking about the frequency of engaging in activities that require a moderate level of energy such as gardening, cleaning the car, or going for a walk. Answers were categorized in a 4-point Likert scale ranging from 1 (more than once a week) to 4 (hardly ever, or never). Finally, quality of life was assessed with the SHARE version of the Quality of Life Scale (CASP-19) [45,46]. Items were answered in a 4-point Likert scale, ranging from 1 (never) to 4 (often), and a total score of quality of life was obtained by summing all item scores. Values ranged from 12 to 48, and higher values indicate better quality of life.

### 2.3. Statistical Analyses

We used finite mixture models estimated in Mplus 8.3. to find the latent classes underlying depression symptoms. Specifically, we estimated several latent class analyses (LCA). LCA is a person-center rather than variable-center statistical method in which we intend to group people with similar patterns of response in the variables under study (depressive symptoms). LCA tries to find the classes, groups, or clusters that simultaneously maximize between-group heterogeneity and within-group homogeneity [47]. Finite mixture modeling is a top–down approach that describes the distribution of the data using a statistical model with goodness of fit indices. LCA also enables confirmatory, between-group analysis, and combines item response theory (and other) models with LCA including covariates to predict the individuals’ latent class membership, or to model changes over time in the structure of the data. Therefore, it is considered more flexible than descriptive cluster algorithms [48].

Latent class analysis can be used as a purely confirmatory technique when there are bases for a theoretical clustering of the data, or it can be used in an exploratory manner. The aim of the LCA, when used in an exploratory manner, is similar to clustering because it tries to obtain the profiles from the data. Exploratory LCA tries to develop a typology, models with different numbers of classes are estimated, and then the statistical criteria and interpretability of the results are used to decide which model to retain. Among the statistical criteria, the information indexes have been widely used: Bayes Information Criterion (BIC), BIC adjusted for the sample size (ABIC); or the Akaike Information Criterion (AIC). All these information criterion, although with slightly different rationale, indicate a better fit of a model with smaller values. These are based on the likelihood function. When fitting models, it is possible to increase the likelihood by adding parameters, with the risk of overfitting. Both BIC and AIC attempt to resolve this problem by introducing a penalty term for the number of parameters in the model; the penalty term is larger in BIC than in AIC. The adjusted version also considers the sample size. Entropy can also be used to make a decision, with entropy values ranging from 0 to 1 (perfect fit). Values for the entropy of 0.7 and over have been considered adequate. Finally, statistical tests have also been developed to compare a model with the model of one less class (model comparison approach). Two of these tests are available in Mplus: the Lo–Mendell–Rubin test (LMR), and Bootstrapped Likelihood Ratio Test (BLRT). Simulation studies have shown that BIC and BLRT work better than other indexes and statistics to assess the model fit [47,49]. In addition to the statistical criteria, selecting the number of latent classes is in part subjective, requiring theoretical and/or practical considerations. Therefore, and with the considerations of [50] in mind, interpretability of the classes was also considered in model selection. Indeed, as this research is of an exploratory type, interpretability has been considered of paramount importance.

Once the model selection is over, the participants may be included into their most likely class and the differences of latent classes in a number of variables of interest can be studied. In order to accomplish this goal, we analyzed the latent class differences in a number of outcomes with chi-square independence tests and analyses of variance, all of them calculated in SPSS 24.

## 3. Results

### 3.1. Latent Class Analyses

Five LCAs were estimated, from one class (baseline) up to five latent classes. Best fitting solution should be chosen as the one with the lowest information criteria (BIC, ABIC, and AIC), largest entropy value, and statistically significant LMR and BLRT tests. Interpretability of results was also considered. Statistical tests and indexes are shown in Table 1.

Statistical criteria were somehow contradictory, specifically, a lack of convergence between the LMR and BLRT. However, and attending to results by [47], we have given priority to the results of the BRLT test. Additionally, the BIC is also known to work better for this type of model [49]. Bearing all this in mind, the inclusion of three to four classes seems reasonable. Whereas the BLRT pointed out that four classes were better than three classes, the LMR tests and the BIC considered the three classes as better than four. Taking into account that entropy was sufficient for the three classes and that we found the three classes were more interpretable than four, we retained this solution. Class 1 included 11.12% of the sample, with class 2 having 14.21% and class 3 comprising the other 74.67% of the sample. Table 2 also includes the chi-square tests of independence to test for the null-hypothesis that each depression symptom is not related with the classes. All symptoms were significantly related except for depressed mood.

Class 1, labeled as psychosomatic, was characterized by large probabilities of having a depressed mood (0.980), sleep disorders (0.912), irritability (0.858), fatigue/loss of energy (0.990), and tears (0.866), with medium to low probabilities in the reminder symptoms. A total of 11.1% of the sample was classified into this class.

Class 2, labeled as melancholic, was characterized by high probability of having pessimism/hopelessness (0.790), suicidal thoughts (0.717), and feelings of guilt (0.655). Nevertheless, this class also showed medium to large probabilities in all of the other symptoms. This class presents the highest probability in suicide ideas, hopelessness, and guilt, which are the most severe symptoms, where hopelessness and inappropriate guilt are two of the characteristics that DSM-V includes for in the melancholy specifier in major depressive disorder. However, another characteristic that must be present in order to include this specifier is anhedonia, which was higher in Class 3 (anhedonic). The symptom showed, however, a moderate probability for the melancholic class. The other symptom included in DSM-V for the specifier of melancholy is a lack of reactivity (symptom that is not included in the EURO-D used). A total of 14.2% of the sample was classified into the melancholic class.

Finally, Class 3, labeled as anhedonic, showed larger probabilities of loss of interest (0.761), concentration problems (0.748), and lack of pleasure (0.658), with low to medium probabilities in symptoms like suicidal thought or irritability, but specially with very low probabilities of feelings of guilt (0.018). The lack of pleasure was the most differential symptom for this class, compared to psychosomatic and melancholic classes. Although the symptoms could represent a dysthymia (DSM-IV) or a persistent depressive disorder with pure dysthymic syndrome (DSM-5), these disorders are characterized by the persistence of symptoms for at least two years (one year for children and adolescents), and the questionnaire used does not discriminate in this regard. A total of 74.7% were classified into this class. 

**Figure 1:**
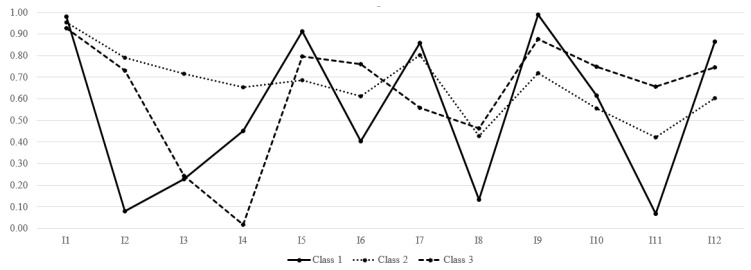
Probabilities of symptoms depending on the class. Notes: y-axis = probabilities; I1 = Depressed mood; I2 = Pessimism; I3 = Suicidal thoughts; I4 = Guilt; I5 = Sleep disorders; I6 = Loss interest; I7 = Irritability; I8 = Appetite; I9 = Fatigue; I10 = Concentration; I11 = Lack pleasures; I12 = Tears; Class 1 = psychosomatic; Class 2 = melancholic; Class 3 = anhedonic.

All probabilities of the three classes in the symptoms are shown in Table 2 as well as graphically shown in Figure 1.

### 3.2. Relations with the Latent Classes

Once the latent classes have been established and people are classified into their more likely class, we can relate classes with variables of interest in order to understand if the different profiles may produce different patterns of relationships. First, classes were related with quantitative variables. An ANOVA on age found significant differences in age between the three classes. A post hoc test found mean differences between the psychosomatic class and anhedonic class, with the psychosomatic class being significantly younger. Perceived health did not show significant mean differences among classes, but there were mean differences in the number of chronic diseases. Post hoc tests showed that the psychosomatic class had significantly less chronic diseases than the anhedonic class (*p* < 0.05). Finally, there were mean differences in quality of life among the three classes, with the psychosomatic class having significantly higher quality of life than the other two classes (*p* < 0.05). ANOVAs and mean values for all classes in all variables are shown in Table 3.

Regarding gender, a chi-square of independence test was not statistically significant (*χ^2^*(2) = 5.227, *p* = 0.073, *V*= 0.092). Widowhood had a significant relation with the latent classes, with more widows or widowers than expected in the anhedonic class and less in the psychosomatic class. People living with a partner were more likely to be in the anhedonic class and less in the psychosomatic class. Mobility also had a significant relation with the classes. There were more people with mobility problems than in the other classes, but nevertheless, mobility problems were very frequent in all classes. When sport activities were considered, again the chi-square was significant. The psychosomatic class exercised more frequently than the other two, although exercising was quite infrequent in all classes. The class that received less help outside their homes was the psychosomatic class, followed by melancholic and anhedonic classes, but nevertheless, in all classes, more than 50% of the people had no help at all. Chi-square tests and percentages within each class are presented in Table 3.

## 4. Discussion

The aim of this research was to estimate the underlying profiles of depressive symptoms among Spanish old adults with a relative high ratio of depressive symptoms, and see how these profiles related to variables associated with depression. Therefore, we tried to understand if there were different associations depending on the particular profile a person with symptoms may be classified.

A routine of several LCAs with a different number of classes was performed to answer this first aim to classify Spanish adults with depression symptoms. Results pointed out the presence of three different classes among the participants in the study. Although in the scientific literature there is a variety of “clustering” solutions with models finding from two up to seven different classes, most research has found three classes to be the best solution [8], which is in line with the current results. With regard to the three classes found, ours were similar to those found by [5] and [10]. The psychosomatic class may be assimilated to negative effect and somatic symptoms [5], or the class with depression with psychosomatic symptoms in [10]. It is, therefore, a type of depression where fatigue and sleep disorders prevail, the latter being one of the main reasons to seek professional help. The etiology of sleep problems is obviously multifactorial, but it should be borne in mind that numerous events occur at this vital stage that may be affecting them such as retirement, change of address, loss of family members, physical illnesses, etc. [51]. Melancholic class is similar to the class found by [5], with a high ratio of symptoms present, or the class of major depression by [10]. It also resembles the melancholic MDD referenced in the DSM-5, which usually occurs in more severe episodes of the disorder [52]. Finally, the anhedonic class is compatible with the class of low symptomatology found by [5], or the class of minor depression in [10]. It is in this last class where concentration problems are more likely, in line with what the DSM-5 [52] has pointed out, and special attention should be paid to this symptomatology (inability to think, to concentrate, ease of distraction, or complaints about problems of memory), since it can sometimes be the initial presentation of irreversible dementia. In addition, it presents a profile of symptoms very similar to persistent depressive disorder, except for the temporality of the symptoms, for which there are no data.

Changes in appetite were not a defining symptom in any of the classes, as though it were critical symptoms for studies [24] and [25]. They were, however, more prominent in melancholic and anhedonic. This could be due to the assessment of appetite with a general item, limited to changes in it, instead of differently assessing increased and decreased appetite or weight. This is an inherent limitation of the EURO-D, and could have affected the results, with other studies pointing the LCA classes were primarily based on weight/appetite differences [18,25]. In any case, the inclusion of symptoms such as increased/decreased appetite in the elaboration of latent classes has recently been criticized by [53], since loss and gain of appetite are considered to be dependent symptoms (who gains appetite and does not lose it) and, therefore, they can generate methodological artifacts based on violations of conditional independence in the latent classes. Further research on the latent classes of depression, taking into account this limitation, would be welcome. 

Regarding the second aim, on one hand, we may observe in the associations found that the psychosomatic class is characterized by being younger, not being widowed, and not living with a partner, exercising more, presenting fewer health problems (chronic diseases and mobility problems), and a higher quality of life compared to the other classes. These characteristics are associated more with the first subtype presented by [5] (low presence of symptoms), than with the subtype of negative affect and somatic symptoms, to which we had equated it. In addition, they receive less social support, which is a more representative result of the subtype with the high presence of symptoms [5]. The melancholic class is characterized by presenting a lower quality of life than the psychosomatic class and anhedonic class, in line with the results presented by [34], and in the rest of the variables, it is at an intermediate point between the psychosomatic class and anhedonic class, or does not differ from them. The anhedonic class was the oldest, the one with more widows or widowers, living in a higher percentage with a partner, and presenting more health problems (chronic diseases and mobility problems), in contrast to the results found by [5] in a similar subtype. In addition, they had a worse quality of life compared to the psychosomatic class [37], but because they had greater social support, perhaps related to a greater need based on these results, the subtypes raised by [5] cannot be assimilated to those found in the present study. This may be because an undiagnosed sample was used in the present study.

On the other hand, no relation was found between class and gender, coinciding with some studies [10,30], nor between class and perceived health, unlike what has been found in the literature [31,32]. Thus, the main results are in line with the scientific literature, affirming that exercise and not being a widower improves or is associated with lower depressive symptomatology, and that at an older age, more chronic diseases and more mobility problems worsen depressive symptoms [33,34,35,36,37]. However, people with a higher quality of life (psychosomatic class) showed that they received less help outside their home, in contrary to what the literature has found so far, with greater social support being associated with less depressive symptoms [34]. This may be because the indicator used to measure social support presents different interpretations such as being younger and with fewer health problems (chronic diseases and mobility), and because of that, they need less help from others outside the home. Additionally, and related to the melancholic class, it should be noted that suicide is directly correlated with age. This is in line with previous studies, which pointed out that higher rates of depression in older adults compared to other periods of life, may be a consequence of the co-occurrence of depression with physical illnesses, decreased impulse control, coupled with cognitive decline, social isolation, and/or loss of spouse [54].

Limitations of the study include, first, the wide heterogeneity of results found in the scientific literature on the LCA of depressive symptomatology, together with scarce evidence in older people. Second, the use of data obtained from the variable aid received from others outside the home to measure social support, instead of using validated scales for older adults such as the Multidimensional Scale of Perceived Social Support (MSPSS) [55], could have given different and richer information than the one obtained. Third, the use of the EURO-D scale instead of the DSM-V or other international classification criteria. A fourth limitation is that the temporality of the symptoms was not taken into account, which may be a variable look in the future, since it can suppose greater or lesser severity of the symptoms as well as characterize a certain subtype or class of depression. Possible future lines of research could include the study of the relationship of the classes with other variables such as age of onset of MDD, monthly family income, number of hospitalizations, or level of metabolic health. Yet another line of research could be to look for profiles of depressive symptoms by gender using LCA, as has been done in other studies [20,56], or exclusively studying one of the genres such as the study by [57] to elucidate whether there were gender-specific subtypes of depression in old people. This would allow us to be more specific and effective in preparing prevention and intervention programs. These studies could also benefit from using biological measures to derive stratifications, as pointed out by [29]: brain-behavior mappings will facilitate clinical phenotypes to be investigated along the axes of variation, allowing targeted treatments to individual patients.

Additionally, potential further investigations may be done by relating the classes found in this sample with data regarding the psychosocial and ecological environment in which people live. SHARE has information (indicators) on social variables such as social connectedness, participation, social network etc., and also has some indicators about the ecological environment in terms of type of town/city, type of building the people live, or a short assessment of the neighborhood. As one reviewer noted, the recent COVID-19 crisis has demonstrated that environmental aspects of our lives may greatly affect not only physical health, but also our mental health, quality of life and wellbeing, just thinking of living in nursing homes, or the type of house or the city/town you live in. Therefore, all these aspects should be further investigated using the typologies of depression found in this and other studies.

In a nutshell, this work represents a step forward to understand the heterogeneity of MDD, with the aim of facilitating prevention at the primary, secondary, and tertiary levels. Regarding primary prevention, this study adds evidence of the importance of reducing the risk of new cases of MDD by intervening in risk factors and the protection of older adults, which could include, for example, the promotion of physical exercise and social support. Regarding secondary prevention, early detection in the primary care of these profiles in the elderly population can facilitate the referral to the corresponding professionals, reducing the waiting time for treatment. With regard to tertiary prevention, the identification of symptom patterns in this specific population can be useful in both applying treatments and preventing more adjusted relapses. Indeed, professionals will be able to take into account both the specific characteristics of older adults with the different types of depression and the relationship observed between the types of depression and some of the related variables, offering guidance on which areas are expected to find lower levels in each specific case of depression to intervene in greater depth. It is, therefore, a study that may have important implications for psychologists and other mental health professionals in their professional practice, since the health resources are limited and the profiles obtained can help to apply these resources in a more efficient way.

## Figures and Tables

**Table 1:** Model fit for 1 to 5 classes.

#classes	AIC	BIC	ABIC	Entropy	LMR Test	*p*	BLR Test	*p*
1	8258.827	8311.828	8273.731	NA	NA	NA	NA	NA
2	8163.057	8273.475	8194.106	0.561	120.328	= 0.057	121.77	< 0.001
3	8122.544	8290.380	8169.738	0.707	65.725	= 0.030	66.513	< 0.001
4	8093.859	8319.112	8157.198	0.738	54.037	= 0.056	54.685	< 0.001
5	8083.220	8365.891	8162.704	0.803	36.204	= 0.015	36.638	< 0.020

Notes: AIC = Akaike Information Criterion; BIC = Bayesian Information Criterion; ABIC = Adjusted BIC; LMR = Lo–Mendell–Rubin test; BLRT = Bootstrapped Log-likelihood Ratio Test; NA = Not applicable.

**Table 2:** Probability of the symptoms in each class and the chi-square differences.

Items	Class 1 (Psychosomatic)	Class 2 (Melancholic)	Class 3 (Anhedonic)	*χ^2^*	*p*
1. Depressed mood	0.980	0.953	0.927	3.14	0.207
2. Pessimism/hopelessness	0.080	0.790	0.731	147.5	<0.001
3. Suicidal thoughts	0.229	0.717	0.245	80.63	<0.001
4. Feelings of guilt	0.452	0.655	0.018	420.5	<0.001
5. Sleep disorders	0.912	0.686	0.797	19.01	<0.001
6. Loss of interest	0.403	0.611	0.761	44.78	<0.001
7. Irritability	0.858	0.801	0.560	44.01	<0.001
8. Changes in appetite	0.135	0.427	0.464	35.14	<0.001
9. Fatigue	0.990	0.720	0.876	33.43	<0.001
10. Concentration problems	0.615	0.557	0.748	19.31	<0.001
11. Lack of pleasure	0.070	0.421	0.658	102.3	<0.001
12. Tears	0.866	0.604	0.745	17.45	<0.001

**Table 3:** Analyses of variance, chi-squares, mean values, and percentages within each class depending on external variables.

	**ANOVAs**				**Psychosomatic Class**				**Melancholic class**				**Anhedonic class**			
	***F***	**df**	***p***	***η^2^***	**M**		**SD**		**M**		**SD**		**M**		**SD**	
Age	14.803	2.609	<0.001	0.046	71.222		8.082		74.712		8.829		77.126		8.829	
Perceived health	2.594	2.609	0.076	0.008	1.89		0.73		1.90		0.87		1.73		0.77	
No. of chronic diseases	12.458	2.608	0.032	0.011	1.51		1.185		1.99		1.435		1.96		1.346	
Quality of life	9.242	2.570	<0.001	0.031	30.40		6.237		27.32		5.519		27.53		5.221	
	**Chi-square**				**Women**		**Men**		**Women**		**Men**		**Women**		**Men**	
	***χ^2^***	**df**	***p***	***V***												
Gender	5.227	2	0.073	0.092	80.9%		19.1%		64.4%		35.6%		72.4%		27.6%	
					**No**		**Yes**		**No**		**Yes**		**No**		**Yes**	
Widowhood	12.633	2	0.002	0.145	82.8%		17.2%		76,7%		23.3%		64.1%		35.9%	
Living with partner	14.622	2	0.001	0.155	76.5%		23.5%		69.0%		31.0%		55.4%		44.6%	
Mobility problems	24.331	2	0.001	0.200	41.2%		58.8%		45.3%		54.7%		23.0%		77.0%	
Received help	13.187	2	0.001	0.147	88.2%		11.8%		79.3%		20.7%		69.1%		30.9%	
					**1**	**2**	**3**	**4**	**1**	**2**	**3**	**4**	**1**	**2**	**3**	**4**
Sports practice	19.414	6	0.126	0.004	19.1%	5.9%	10.3%	64.7%	11.5%	4.6%	2.3%	81.6%	6.8%	3.5%	5.3%	84.5%

*Note:* 1 = More than once/week; 2 = Once/ week; 2 = 1–3 times/ month, 3 = Never or almost never.
