# Erratum: Pérez-Belmonte, S., et al. Subtypes of Depression: Latent Class Analysis in Spanish Old People with Depressive Symptoms. *Life* 2020, *10*, 70

**DOI:** 10.3390/life10100222

**Published:** 2020-09-27

**Authors:** Sergio Pérez-Belmonte, Laura Galiana, Patricia Sancho, Amparo Oliver, José M. Tomás

**Affiliations:** 1 Department of Methodology for the Behavioral Sciences, University of Valencia, 46010 Valencia, Spain; Sergioperezbel@gmail.com (S.P.-B.); oliver@uv.es (A.O.); tomasjm@uv.es (J.M.T.); 2 Department of Educational and Developmental Psychology, University of Valencia, 46010 Valencia, Spain; Patricia.Sancho@uv.es

The authors wish to make the following erratum to this paper [1]. The Funding is incorrect and must be replaced by the following:

This research was funded by project (RTI2018-093321-B-I00) supported by: ERDF (European Regional Development Fund), Ministry of Science and Innovation and State Research Agency from Spain.

The authors would like to apologize for any inconvenience caused to the readers by these changes. The *Life* editorial office has been asked to update the original manuscript [1] on the article webpage, with a reference to this erratum.
